# Analysis of recent segmental duplications in the bovine genome

**DOI:** 10.1186/1471-2164-10-571

**Published:** 2009-12-01

**Authors:** George E Liu, Mario Ventura, Angelo Cellamare, Lin Chen, Ze Cheng, Bin Zhu, Congjun Li, Jiuzhou Song, Evan E Eichler

**Affiliations:** 1 USDA, ARS, ANRI, Bovine Functional Genomics Laboratory, Beltsville, Maryland 20705, USA; 2 Department of Genetics and Microbiology, University of Bari, Bari 70126, Italy; 3 Deparment of Genome Sciences, University of Washington School of Medicine, Seattle, Washington 98195, USA; 4 Department of Bioengineering, University of Maryland, College Park, Maryland 20742, USA; 5 Department of Animal and Avian Sciences, University of Maryland, College Park, Maryland 20742, USA; 6 Howard Hughes Medical Institute, Seattle, Washington 98195, USA

## Abstract

**Background:**

Duplicated sequences are an important source of gene innovation and structural variation within mammalian genomes. We performed the first systematic and genome-wide analysis of segmental duplications in the modern domesticated cattle (*Bos taurus*). Using two distinct computational analyses, we estimated that 3.1% (94.4 Mb) of the bovine genome consists of recently duplicated sequences (≥ 1 kb in length, ≥ 90% sequence identity). Similar to other mammalian draft assemblies, almost half (47% of 94.4 Mb) of these sequences have not been assigned to cattle chromosomes.

**Results:**

In this study, we provide the first experimental validation large duplications and briefly compared their distribution on two independent bovine genome assemblies using fluorescent in situ hybridization (FISH). Our analyses suggest that the (75-90%) of segmental duplications are organized into local tandem duplication clusters. Along with rodents and carnivores, these results now confidently establish tandem duplications as the most likely mammalian archetypical organization, in contrast to humans and great ape species which show a preponderance of interspersed duplications. A cross-species survey of duplicated genes and gene families indicated that duplication, positive selection and gene conversion have shaped primates, rodents, carnivores and ruminants to different degrees for their speciation and adaptation. We identified that bovine segmental duplications corresponding to genes are significantly enriched for specific biological functions such as immunity, digestion, lactation and reproduction.

**Conclusion:**

Our results suggest that in most mammalian lineages segmental duplications are organized in a tandem configuration. Segmental duplications remain problematic for genome and assembly and we highlight genic regions that require higher quality sequence characterization. This study provides insights into mammalian genome evolution and generates a valuable resource for cattle genomics research.

## Background

Segmental duplications have been recognized as important mediators of both gene and genome evolution [1-9]. Segmental duplications are substrates of genome innovation, genomic rearrangements, and hotspots of copy number variation (CNV) within species [7,8,10-12]. From the genic perspective, such duplications often encode protein products which, although not essential for viability of the organism, are important for the adaptation of the species to specific ecological niches [13]. Among mammalian species, commonly duplicated genes include those associated with the recognition of environmental molecules and include genes associated with innate immunity, drug detoxification, olfaction, and sperm competition. From the perspective of genome structure, lineage-specific segmental duplications or large repeats often delineate regions of recurrent evolutionary liability [2,7,14]. Recent comparative sequencing efforts among mammals, for example, shows that highly homologous repetitive sequence frequently associate with the breakpoints of large-scale chromosomal rearrangement [15-18]. Understanding the nature and pattern of segmental duplications provides fundamental insight into functional redundancy, adaptive evolution, and the structural dynamics of chromosomal evolution [7,8,19-25]. From the practical perspective, regions of large-scale duplication are particularly problematic for genome assembly, SNP mapping and genotyping [1,26-28]. For example, two independent bovine genome assemblies were recently reported (Btau_4.0 and UMD2) and a simple comparison indicated that significant fewer intrachromsomal duplications were detected in UMD2 [29,30]. Gene and SNP annotation will significantly improve when duplicated sequence is correctly integrated into the assembly [31,32]. Knowledge of the location and content of duplicated regions could be important for accurately mapping QTL, and validating putative single-nucleotide polymorphisms (SNPs) that may have arisen from allelic variants as opposed to recently duplicated sequences[33].

Segmental duplications have been extensively studied in other organisms [1,2,4,7,8]. Here we report in detail our genome-wide and systematic analysis of segmental duplications in cattle using Btau_4.0. We further validated the distribution of selected large duplications and briefly compared their corresponding regions in Btau_4.0 to a second bovine assembly, UMD2, using FISH. We performed a cross-mammalian survey of duplicated genes and gene families to compare gene repertoires and evolutionary mechanism of origin. Along with rodents and carnivores, our bovine results now establish tandem duplications as the most likely mammalian archetypical organization, in contrast to higher primates which show a preponderance of interspersed duplications.

## Results

### Genome-wide Identification of Bovine Segmental Duplications

**Figure 1:**
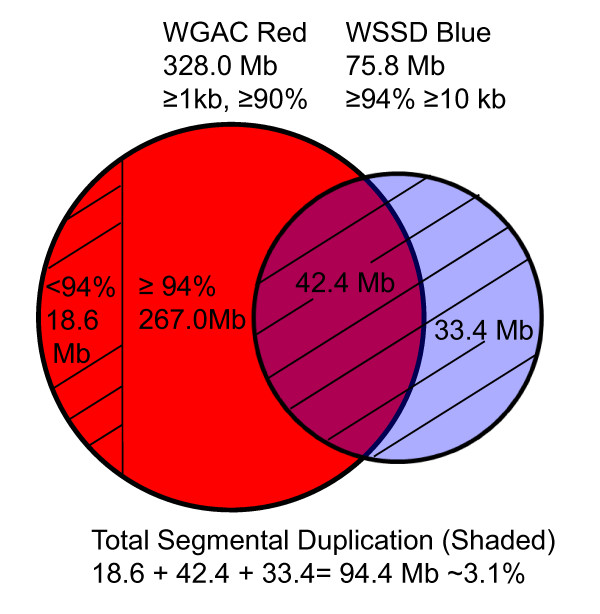
**Comparison of bovine segmental duplications predicted by the WGAC and WSSD algorithms**. We initially identified 328.0 (red) and 75.8 Mb (blue) as putative duplications using the WGAC and WSSD analysis, respectively. The overlapping relationship of these two predictions is shown in a Venn diagram. We defined segmental duplications based on the union of significant WGAC hits with less than 94% sequence identity (18.6 Mb, shaded red) and WSSD results (75.8 Mb, shaded blue).

We applied two well-established computational approaches, whole genome shotgun sequence detection (WSSD) [1] and Whole Genome Assembly Comparison (WGAC) [34], to the publicly available bovine genome sequence assembly (Btau_4.0) to detect putative segmental duplications. Briefly, WGAC identifies paralogous sequences ≥ 1 kb in length with ≥ 90% sequence identity, while the WSSD identifies genomic regions that exhibit significant depth of coverage by aligning whole genome shotgun sequencing reads to the reference genome sequence (≥ 10 kb, ≥ 94%). Remarkably, we initially identified 328.0 Mb or 129,555 pairwise alignments as putative duplications by the WGAC analysis (47,261 map to unassigned scaffolds - ChrUnAll). Of the 10,251 intrachromosomal (scaffolds assigned to chromosomes) segmental duplications, 71% (n = 7,245) map within 1 Mb of one another. As larger, high-identity duplications (the 267.0 Mb unshaded region in Fig. 1) are frequently collapsed within working draft sequence assemblies [28] or may represent artificial duplications within an assembly [34], we compared these WGAC results to those detected by the assembly-independent WSSD approach. We found that 44% of the WSSD duplication intervals (33.4/75.8 Mb) were not detected by the genome assembly based comparison and, likely represent collapsed duplications (Fig. 1). In addition, we identified 42.4 Mb high-confidence duplications detected by both methods. These include 30,559 pairwise alignments (14,207 interchromosomal and 16,352 intrachromosomal).

We note the presence of a large fraction of sequence (92.5 Mb) detected by WGAC (≥ 20 kb, ≥ 94% identity) from the 267.0 Mb regions as defined above with no WSSD overlap. Excluding the unassigned scaffolds, these are predominantly intrachromosomal in origin and a total of 364/402 (91%) pairwise alignments map within 1 Mb of one another. As large, high identity alleles (≥ 99.5%) may not be merged and represent artificial duplications due to local assembly errors [34], we excluded all these alignments to eliminate artificial duplications in Batu_4.0. Our results are also supported by the observation reported by Zimin et al [30] that overwhelming majority of the large, high-identity intrachromosomal duplications (> 5 kb, > 95%) are probably assembly artifacts. Their brief duplication analysis indicated that Batu_4.0 had significantly more duplications of this type, 3,098 vs. 662 in UMD2.

**Figure 2:**
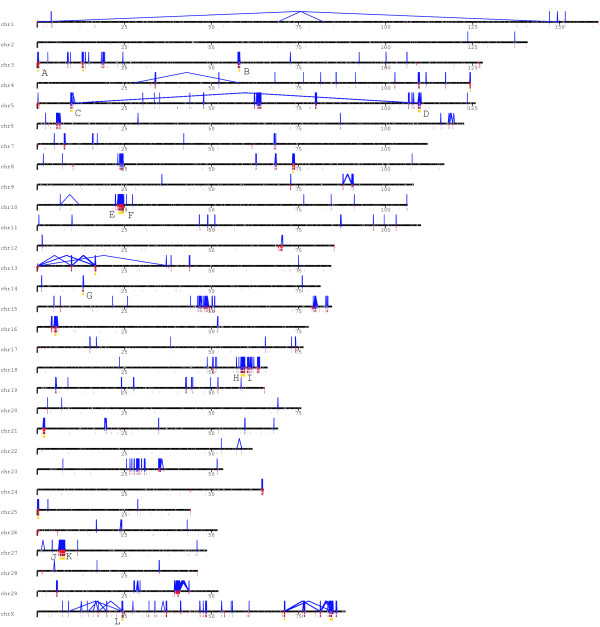
**Bovine segmental duplication landscape (≥ 5 kb in legnth, ≥ 90% sequence identity)**. Intrachromosomal (blue, with connecting lines) and interchromosomal (red bars, without connecting lines) duplications are shown on Batu_4.0. White bars represent gaps in the genome assembly. A local tandem distribution pattern is predominant in bovine segmental duplications. With few exceptions, most intrachromosomal duplications are organized as clusters of tandem or inverted duplications within close proximity (1 Mb). A total of 21 large regions (each ≥ 300 kb in length, total ~12.6 Mb of sequence) are shown as gold bars. Twelve of these duplication blocks (labeled A to L) correspond to known genes (A:LAD1; B:GBP6; C:WC1.1, WC1.2, CD163L1, SYT1; D:WC1.3; E: T-cell receptor alpha clusters; F: T-cell receptor delta clusters; G:ANKRD26, FBXO18; H:Zinc finger protein clusters, ACTR2; I: Zinc finger protein clusters; J: β-defensins 2, 4,7,8, and 10; K: β-defensins 1 and 5; L: VAMP7). For more detail, including sequence identity and pairwise relationships of all duplications and alignments, see http://bfgl.anri.barc.usda.gov/cattleSD/. For details about patterns of interchromosomal duplications, see Additional file 1: Fig. S1.

Following our previous studies of other genomes, we defined segmental duplications based on the union of all WGAC hits with less than 94% sequence identity and WSSD duplication intervals (Fig. 1). We derived an estimate of the duplication content of the bovine genome to be 3.11% (94.4 Mb/3,036.6 Mb; Fig. 2 and also see Additional File 1: Fig. S1). This, however, should be regarded as conservative estimate that will increase as the bovine genome assembly improves. In the following analyses, we focused on further characterization of this subset.

### Distribution and Sequence Properties of Bovine Segmental Duplications

The recent segmental duplications of the bovine genome are distributed in a nonrandom fashion at two different levels. First, duplication content varies significantly among different chromosomes. Chromosomes 5, 18, 27, 29 and X show the greatest enrichment for segmental duplication (See Additional File 1: Table S2, Fig. S1 and S3) with twofold the duplication content of the genome average (excluding unplaced sequence contigs). Most of this effect is due to an increase in intrachromosomal duplication content localized at specific clusters. Furthermore, similar to the human, mouse, rat and dog genomes, there are a greater proportion of duplications near pericentromeric and subtelomeric regions. Excluding unmapped contigs, pericentromeric regions represent 3.4% of genomic sequence, but show an enrichment of 2.4-fold for duplications (p-value < 0.001) and contain 8.1% of all duplicated bases. Similarly, subtelomeric regions show an enrichment of 1.9-fold (p-value < 0.001) and contain 6.7% of duplicated bases. Additionally, a strong positive correlation between segmental duplication and evolutionary breakpoint regions was observed [29]. As expected, the "uncharacterized chromosome" (ChrUnAll), which consists of sequence that cannot be uniquely mapped to the genome, contains the majority of predicted duplication bases (45.3/94.4 Mb, 47%, see Additional File 1: Fig. S2).

**Figure 3:**
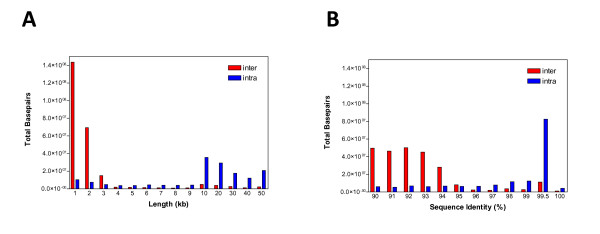
**The distribution of length and percent identity for high-confidence segmental duplication detected by WGAC and WSSD**. Panel A shows the length distributions while panel B shows the pairwise sequence identity distribution for the segmental duplications. Red, interchromosomal segmental duplications; blue, intrachromosomal segmental duplications.

**Figure 4:**
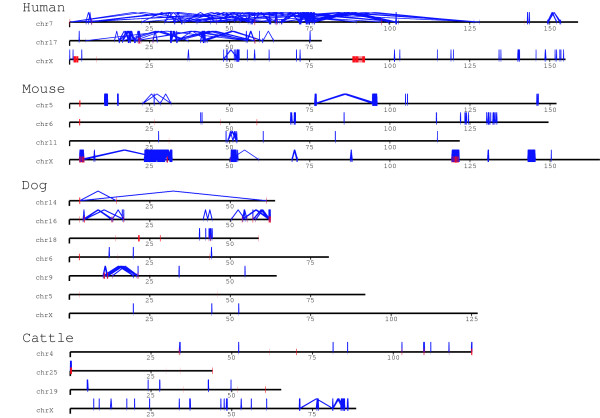
**The intrachromosomal duplication patterns in mammalian genomes: human and mouse (> 20 kb, > 94%) and dog and cattle (> 10 kb, > 94%)**. The human genome displays interspersed pattern of recent duplications as compared to the tandem clusters in the mouse, dog and cattle genomes. Based on UCSC Genome Browser Human Net tracks, chrX is syntenic among these mammals. Human chr17 is syntenic to mouse chr11, dog chr9 and chr5 and cattle chr19. Human chr7 is syntenic to mouse chr6 and chr5, dog chr14, chr16, chr18 and chr6 and cattle chr4 and chr25.

Of those duplications that can be assigned to a chromosome and confirmed by two different duplication algorithms, we note a bipartite distribution with respect to length and percent identity (Fig. 3). Fig. 3 depicts the duplication content of the bovine genome as a function of the length of alignment and the degree of sequence identity. Interchromosomal duplications are shorter (median length 2.5 kb) and more divergent (< 94% identity), while intrachromosomal duplications are much larger (median length 20 kb) showing higher sequence identity (~97%). There are 1,020 duplication intervals from duplicated sequence identified by WGAC and WSSD with a median length and average length of 48.8 kb and 82.8 kb, respectively. Twenty-one of these duplication blocks are ≥ 300 kb in length and located in regions enriched in tandem duplications, including multiple known gene clusters (Fig. 2 and Additional File 1: Fig. S1). This pattern is reminiscent of the duplication pattern of other mammals (mouse, rat and dog) but differs from the interspersed segmental duplication pattern that predominates in human and great-ape genomes (Fig. 4) [1,3,4,7,8,20].

Delineation of the most recent duplication events at the genomic-sequence level, and particularly sequences located at their junctions [35], may provide insight into their mechanism of origin [15,17,36,37]. We compared the repeat content of duplicated sequence, flanking sequence and the whole genome (Table 1, Methods). Unlike human segmental duplications, which are enriched for SINE *Alu *repeats [35], no SINE enrichment was associated with bovine segmental duplications. The working draft nature of the bovine genome sequence currently prevents a detailed analysis of the sequence structure at the transition regions between unique and duplicated sequence. Nevertheless, two clear patterns emerge regarding repeat content. While LTR content remains similar, DNA, SINE and low-complexity repeat content of most duplications are reduced (Table 1, Random simulation test, P-values < 0.001). SINE content shows a reduction compared to the genome average (12.71% vs. 15.90%). This gradually increases to the genome average as sequences flanking the duplications are considered. An opposite trend is observed with respect to LINE and satellite repeat sequences, even though the fold change for LINE is only 1.03 (P-value < 0.01). Compared to the genome average, bovine segmental duplications show a 1.91-fold enrichment for satellite repeat content and a 2.24-fold elongation for satellite repeat average length (Table 1). When individual repeat subfamilies are considered, two related satellite repeat classes BTSAT4 and OSSAT2 show the greatest increases in length and/or density while BTSAT2 and BTSAT6 show decreases in both length and density. Satellite BTSAT4 shows 2.26-fold increase in density while their average lengths are similar. Satellite OSSAT2 shows 7.55-fold increase in density and 2.71-fold increase in length.

**Table 1:** Repeat properties of bovine genome, duplications, and flanking regions

Repeat	Duplications					20-kb flanks					Genome					Enrichment ratio in duplication content		
	
	Length	Count	Ave. Len.	Length%	Count/ 1 Mb	Length	Count	Ave. Len.	Length%	Count/ 1 Mb	Length	Count	Ave. Len.	Length%	Count/ 1 Mb	Length%	Count/ 1 Mb	Ave. Len.
**DNA**	505,659	2,777	182	1.09%	6.01	281,144	1,599	176	1.62%	9.20	56,383,709	304,851	185	1.93%	10.45	0.57**	0.58	0.98
**LINE**	10,138,094	23,014	441	21.95%	49.82	3,993,714	10,320	387	22.99%	59.40	624,600,656	1,655,642	377	21.41%	56.74	1.03*	0.88	1.17
**SINE**	5,870,448	26,627	220	12.71%	57.64	2,699,548	12,975	208	15.54%	74.68	464,030,104	2,203,064	211	15.90%	75.50	0.80**	0.76	1.05
**LTR**	1,565,238	6,322	248	3.39%	13.68	661,698	2,893	229	3.81%	16.65	99,886,031	444,168	225	3.42%	15.22	0.99	0.90	1.10
**Satellite**	143,866	49	2,936	0.31%	0.11	22,946	53	433	0.13%	0.31	4,749,093	3,626	1,310	0.16%	0.12	1.91*	0.85	2.24
**Simple**	214,338	4,972	43	0.46%	10.76	88,930	1,999	44	0.51%	11.51	14,261,600	343,825	41	0.49%	11.78	0.95	0.91	1.04
**Low** **complexity**	169,741	4,390	39	0.37%	9.50	80,640	2,050	39	0.46%	11.80	14,280,594	370,243	39	0.49%	12.69	0.75**	0.75	1.00
**Other**	100,574	1,263	80	0.22%	2.73	63,019	793	79	0.36%	4.56	8,582,076	105,321	81	0.29%	3.61	0.74	0.76	0.98
**Total** **repeat**	18,707,958	69,414	270	40.50%	150.26	7,891,639	32,682	241	45.42%	188.11	1,286,773,863	5,430,740	237	44.10%	186.11	0.92	0.81	1.14
**Total** **analyzed**	46,196,844	138,828				17,373,955	65,364				2,917,958,192	10,861,480						

We compare the repeat content of duplicated regions as detected by both WSSD and WGAC (with ChrUnAll excluded); 20-kb flanking regions immediately flanking the clustered duplications and the genome average. Enrichment ratios were defined as the total repeat length, count/1 Mb and average length of duplicated sequence divided by the repeat length, count/1 Mb and average length of genome. The significance of the enrichment was determined by simulating the repeats in a random sample (*n *= 1,000) of cattle duplicated genomic sequence (**: P-value < 0.001, *: P-value < 0.01).

### Gene Content

We considered the genomic duplication content of the gene sets aligned to the bovine genome. Seventy-six percent (778/1020) of the bovine segmental duplication intervals identified by both WGAC and WSSD correspond to complete or partial gene duplications (See Additional File 1: Table S3). Of these, the overwhelming majority of pairwise alignments was < 1 Mb apart, again indicating that most "functional" duplicates within the bovine genome are clusters of tandem gene families, as opposed to widely interspersed duplications in humans and other primates. Although a portion of these intervals correspond to predicted genes of unknown or hypothetical function, 1,858 RefSeq genes were located in predicted segmental duplications. In order to test the hypothesis that particular gene classes are overrepresented in duplicated regions, we assigned PANTHER Molecular Function terms to all genes that overlapped duplications. Statistically significant over or under representations were observed for multiple categories (Additional File 1: Table S4). Another independent Gene Ontology and pathway analyses also confirm that these terms and categories are significantly enriched in bovine segmental duplication regions [29].

Consistent with similar duplication analyses in other mammals [1,3,4,7,10], several of these gene duplications, which are important in drug detoxification, defense/innate immunity and receptor and signal recognition, are also duplicated in cattle (such as cytochrome P450, ribonuclease A, and β-defensins). Since these genes or gene families have been repeatedly detected to be duplicated in multiple mammalian genomes, it will be interesting to investigate their repertoires and evolutionary mechanisms. Combining the bovine gene annotation effort [29], our duplication analyses and other published results, we surveyed and summarized the evolutionary analyses of 7 well-studied duplicated gene families in cattle, humans, mice and dogs (Table 2). These multiple-member gene families normally went through the so-called "birth-and-death" evolution [38] in which new genes were created by gene duplication and some of them were retained in the genome for a long time as functional genes, but other genes were inactivated or eliminated from the genome. While some ancient members arose before the last common ancestor of mammals, a common theme is that new members often originated after divergence of these mammals from each other. These lineage-specific gene expansions of individual subfamilies were detected in all 4 species, especially in cattle and mice.

**Table 2:** Repertoires and evolutionary mechanisms of selected duplicated genes or gene families in mammals

	Bovine	Human	Mouse	Dog	Mechanisms
Cytochrome P-450	~65	57	102	54	Duplication, inactivation; positive selection [43]
Ribonuclease A	21	13	25	7	Duplication, deletion, inactivation; positive selection and alternative splicing [39]
β-Defensins	~106	39	52	43	Duplication, deletion, inactivation; positive selection [29,41,42]
IFNA	13	13	14	9	Duplication, deletion, inactivation; no strong positive selection but strong gene conversion [44,45]
Olfactory Receptor	1152	388	1063	822	Duplication, deletion, inactivation; positive selection [40]
TCRV*	170	111	78	41	Duplication, deletion, inactivation; positive selection [46]
C-type Lysozyme	10	1	3	2	Duplication; positive selection [29]

As many of these duplicated genes are present in unassigned chromosomes, gene numbers in the bovine genome are based on the cited literature and some of them are just best estimates. *Based on searches on the international ImMunoGeneTics (IMGT) information system at http://www.imgt.org/.

Depending on their nature (gene ancestries, structures, functions, and genomic distributions), three major evolutionary mechanisms - gene duplication, positive selection and conversion have shaped these gene families to different degrees. For example, phylogenetic analysis of RNase A indicates that this gene family expansion predated the separation of placental and marsupial mammals and that differential gene duplication and loss occurred in different species, generating a great variation in gene number and content among extant mammals [39]. Similarly, gene duplication and inactivation have important roles in both the adaptive and non-adaptive evolution of Olfactory Receptor (OR) genes [40]. Another example is β-defensin genes which are densely clustered in four to five syntenic chromosomal regions. Although the majority of β-defensins are evolutionarily conserved across species, subgroups of gene lineages exist that are specific in species like cattle and mice and originated recently by gene duplication and positive selection [29,41,42]. An analysis of cytochrome P450 gene families in 10 vertebrate species provided two distinct evolutionary schemes depending on gene functions. While stable genes for endogenous metabolic functions are characterized by few or no gene duplications or deletions, unstable genes for xenobiotics detoxification are characterized by frequent gene duplications, deletions and positive selection [43]. Finally, gene conversion has played a major role in shaping the *IFNA *gene family in eutherian species after gene duplication [44,45]. The other duplication examples include *TCRV *[46], C-type lysozymes [29], *BPI-like *(*BSP30*) [47], *BPI*/*LBP*, Cathelicidin [29], interferon subfamilies (*IFNB*, *IFNW*, and *IFNX*) [29], Pregnancy-associated glycoprotein [48], Sulfotransferases, *ULBP *[49], *WC1 *[50] and etc.

**Figure 5:**
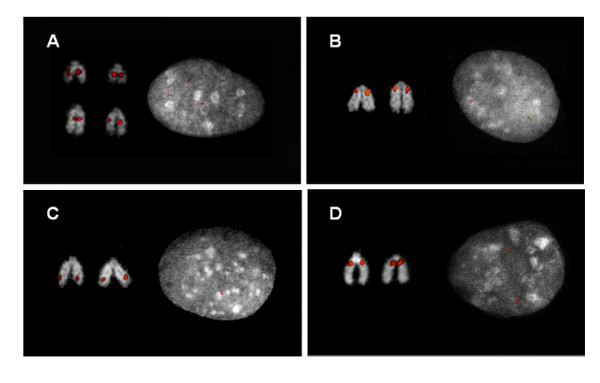
**FISH confirmation**. Examples of metaphase and interphase FISH hybridization with duplicated BAC clones and their associated genes. A. 154H9 (LYZ1: lysozyme 1), B. 170G20 (PDE5A: cGMP-specific phosphodiesterase 5A), C. 27N13 (STX7: syntaxin 7), and D. 303B2 (ZFP2: zinc finger protein 2 homolog). The results of all FISH experiments are available online at http://bfgl.anri.barc.usda.gov/cattleSD/.

The high level of sequence identity (median = 98.9%) indicates that over 25% (263/1020 > 99.0%) of the bovine duplications may have occurred within the artiodactyla, and probably more specifically within the *Bos *lineage. For example, some genes are only duplicated in cattle but not in other mammalian lineages (eg. *matrilin*, *conglutinin*, *CBX3*, *CSKN1B*, etc in 2 Additional File 1: Table S3). Additionally, some of these may represent gene families important in cattle adaptation or recent domestication. There are also considerable gene duplications involved in adaptive immune responses in cattle compared with human and mouse. For example, we detected 2 duplication blocks containing at least 13 *WC1 *genes distributed within two distant regions on chr5 (Fig. 2). We find evidence of recent duplication of the intelectin gene (*ITLN1; *lactoferrin receptor), which is a receptor for a major iron-binding protein in milk, and a sterol carrier protein (*SCP2*), which is an intracellular protein potentially involved in lipid transfer in organs involved in lipid metabolism, including mammary tissue. Two other genes encoding proteins present in milk during lactation or mastitis were found to be associated with segmental duplications: cathelicidin (*CATHL1*) and β-2 microglobulin (*B2M*). In addition, there is over-representation of genes involved in ruminant-specific aspects of reproduction including the intercellular signaling proteins pregnancy associated glycoproteins (chr29), interferon tau (*IFNT *on chr8), trophoblast Kunitz domain proteins (chr13) and prolactin-related proteins (chr23). Our predictions and FISH results also confirmed that the expansion of the well-known C-type lysozyme family though gene duplication (described below and Fig. 5).

### FISH Characterization of Predicted Segmental Duplication and Comparison of Bovine Genome Assemblies

We experimentally validated a subset of the largest (≥ 20 kb) duplicated regions by FISH (Fig. 5). Forty-six large-insert BAC clones identified by WGAC and/or WSSD methods were used as probes and hybridized against a Hereford smooth muscle cell line (Tables 3 and 4). One BAC clone (210P15) was only supported by WGAC < 94% (i.e. from the 18.6 Mb region of Fig. 1). Six of the probes failed to generate signals by hybridization. We observed multiple signals either by examination of interphase and metaphase FISH for 80% (33/41) of the remaining probes confirming their duplication status including duplications of cattle genes of *ALAS2*, *BCAS2*, *GEMIN8*, *LDB2*, *MED6*, *NMT2*, and *ZFP2*. As expected, the majority (72%) of the WSSD intervals without assembly support for duplications (WGAC negative) were confirmed by FISH. Only 2 of the probes showed signals on non-homologous chromosomes (interchromosomal duplications). 93% of the probes (31/33) showed evidence of duplicated signals that were locally clustered (tandem intrachromosomal duplication). 16 out of 33 duplicated BACs produced pericetromeirc or telomeric signals. The BAC probes (154H9) covering *LYZ1 *produced duplicated signals both interchomosomally and intrachomosomally (Dup inter and Dup intra). Similar to the mouse and dog genomes [7,8], these FISH data demonstrate that tandem intrachromosomal duplications predominate in the cattle genome (Fig. 2 and Fig. 4). The basis for the remaining 8 BAC probes consistent with single copy sequence is unknown. We note, however, that the animal for the cell line used in the FISH experiments is a Hereford male different from the sequenced cow (Dominette), and copy number polymorphism as well as limitations of BAC-FISH to detect duplications < 40 kb (especially in the case of local tandem duplications) may account for differences between the computational predictions and experimental data.

**Table 3:** Confirmation of bovine segmental duplications by FISH analysis

	BAC	FISH	Dup all	Dup intra	Dup inter	Single	No Result
Both	33	29	24	22*	2	5	4
WSSD	12	11	8	8	0	3	1
WGAC < 94%	1	1	1	1	0	0	0
Total	46	41	33	31	2	8	5
Percentage	---	100%	80.49%	75.61%	4.88%	19.51%	---

A set of 46 large-insert BAC clones from CHORI-240 library were selected and independent FISH hybridizations were performed. One BAC clone was only supported by WGAC < 94%. FISH signals were categorized as "Single", "Dup intra" or "Dup inter" based on the presence of a single signal or multiple signals on the same or different chromosomes for each probe. *It does not include 154H9, which was scored as both Dup inter and Dup intra.

**Table 4:** FISH characterization of Predicted Segmental Duplication

No	Type	BAC	Genomic Coordinates	FISH Results	Genes	Location
1	Both	109B15	chr3:31163855-31372136	Dup intra	BCAS2	Pericentromeric
2	Both	508O4	chr3:99917302-100113132	Single	SCP2	NA
3	Both	107O12	chr4:102574988-102800832	Dup inter	SLC23A2	Telomeric
4	Both	232G14	chr5:107677204-107842367	No results	KLRF1	NA
5	Both	230B1	chr5:109254775-109460758	Dup intra	no gene	Telomeric
6	Both	410D11	chr5:40136577-40302384	Dup intra	no gene	Interstitial
7	Both	154H9	chr5:47523133-47830066	Dup inter &Dup intra	LYZ1	Interstitial
8	Both	170G20	chr6:6086819-6350887	Dup intra	PDE5A	Pericentromeric
9	Both	453M23	chr6:6418121-6667425	Dup intra	MGC134093	Pericentromeric
10	Both	303B2	chr7:2221666-2410963	Dup intra	ZFP2	Pericentromeric
11	Both	27N13	chr9:72578833-72767038	Dup intra	STX7	Interstitial
12	Both	460I2	chr10:84431632-84620341	Dup intra	MED6	Interstitial
13	Both	194H8	chr11:46793969-47036106	Dup intra	no gene	Interstitial
14	Both	445I3	chr12:85207002-85379077	Dup intra	Tel	Telomeric
15	Both	162G14	chr13:16282432-16506351	Dup intra	IL2RA	Interstitial
16	Both	512C3	chr13:29199709-29453354	Dup intra	NMT2	Interstitial
17	Both	471B24	chr18:58267050-58503186	Dup intra	ZNF	Interstitial
18	Both	306H19	chr19:40075431-40253104	No results	NM_006310	NA
19	Both	500F22	chr19:42649170-42786581	Dup intra	KRTRP	Pericentromeric
20	Both	277E6	chr27:6257023-6444256	No results	BTN3A3	NA
21	Both	412A22	chr29:8616714-8796296	No results	TMEM135	NA
22	Both	64B3	chrX:17312915-17514658	Dup intra	no gene	Pericentromeric
23	Both	250G12	chrX:24170707-24419693	Dup intra	VAMP7	Pericentromeric
24	Both	213C22	chrX:31835480-32033216	Dup intra	no gene	Pericentromeric
25	Both	13B5	chrX:53583006-53778536	Single	no gene	NA
26	Both	176G5	chrX:56052875-56258742	Dup intra	CSNK1B	Interstitial
27	Both	476D12	chrX:71108517-71271350	Single	MAGEB18	NA
28	Both	431K8	chrX:80676183-80893001	Dup intra	GEMIN8	Telomeric
29	Both	29P20	chrX:85621779-86016472	Dup intra	ZNF	Telomeric
30	Both	190O6	chrUn.004.137:11823-223496	Dup intra	IFITM1	Interstitial
31	Both	272A17	chrUn.004.17:246225-650728	Dup intra	CYSLTR1, MARCH5	Interstitial
32	Both	417D14	chrUn.004.32:368390-592637	Single	ZXDA	NA
33	Both	147N16	chrUn.004.37:2-215660	Single	OR7A5	NA
34	WSSD	437P5	chr5:78220396-78401696	Single	no gene	NA
35	WSSD	154G22	chr6:116004945-116244550	Dup intra	LDB2	Telomeric
36	WSSD	114C16	chr13:10828181-11062504	Dup intra	no gene	Interstitial
37	WSSD	156M23	chr16:50843873-51041572	Dup intra	no gene	Interstitial
38	WSSD	82C14	chr20:3586174-3798411	Dup intra	NM_033644	Pericentromeric
39	WSSD	77E4	chr21:21099409-21300561	Dup intra	no gene	Interstitial
40	WSSD	90O14	chr21:64173842-64394464	No results	no gene	NA
41	WSSD	23F3	chr24:6824971-6986816	Single	no gene	NA
42	WSSD	104N21	chr28:13010789-13201901	Dup intra	no gene	Interstitial
43	WSSD	3M17	chrX:60292098-60448656	Single	ALAS2	NA
44	WSSD	129M19	chrUn.004.1055:2-52361	Dup intra	no gene	Heterochromatin
45	WSSD	400H20	chrUn.004.1612:55-32829	Dup intra	no gene	Heterochromatin
46	WGAC (< 94%)	210P15	chr13:37614994-37864888	Dup intra	SLC6A9	Telomeric

*NA: not assigned. One BAC clones (210P15) was only supported by WGAC < 94%.

As local assembly errors, e.g. artificial duplications, are particularly enriched in large, high-identity duplicated regions identified only by WGAC (the 267.0 Mb unshaded region in Fig. 1), we chose 13 additional BAC clones from that region (99.69% to 99.98% sequence identity) as FISH probes to compare Btau_4.0 and UMD2. Since the BAC-FISH method used here was most reliable to distinguish single signal vs. duplicated signals and interchromosomal (Dup inter) vs. intrachromosomal (Dup intra) duplications, our assembly comparisons were mainly based on these two criteria. Table 5 summaries our comparisons of the FISH results with the computational predications based on Btau_4.0 or UMD2. Except for one BAC clone (11L7) which had no results, 10 BAC clones supported UMD2's predictions while only 2 supported Btau_4.0's. This result is a striking contrast to what we obtained for those 46 BAC clones of Both, WSSD and WGAC < 94% types (Table 4). The two assemblies essentially produced the same computational predictions for those 46 BAC clones (data not shown), suggesting two assemblies are almost identical in those high-confidence duplicated regions.

**Table 5:** FISH comparison of two bovine genome assemblies

No	Type	BAC	Genomic Coordinates	Genes	Location	FISH	Btau_4.0	UMD2	Support
1	WGAC	29M8	chr1:133744175-133954795	no gene	Telomeric	NYN	NNY	NYN	UMD2
2	WGAC	388F24	chr2:132507168-132702161	CLIC4	Telomeric	NYN	NYY	NYN	UMD2
3	WGAC	388H21	chr3:63168745-63555485	SSX2IP	Interstitial	NYN	NNY	NYN	UMD2
4	WGAC	11L7	chr4:68544767-68768755	no gene	NA	NA	NYY	YNN	NA
5	WGAC	419I3	chr5:50034725-50202046	no gene	Interstitial	NYN	NYY	YNN	Btau_4.0
6	WGAC	518J9	chr5:73679193-73890817	APPL2	NA	YNN	NNY	YNN	UMD2
7	WGAC	457K23	chr6:77152427-77315213	no gene	NA	YNN	NNY	YNN	UMD2
8	WGAC	512L10	chr9:99288981-99490065	no gene	NA	YNN	NYY	YNN	UMD2
9	WGAC	526E10	chr12:7697603-7874322	no gene	Pericentromeric	NYN	NYY	NYN	UMD2
10	WGAC	6B15	chr13:47237506-47492899	SLC23A2	Telomeric	NYN	NNY	NYN	UMD2
11	WGAC	42E18	chr19:43869525-44087448	ATP6V0A1	Interstitial	NYN	NYY	NYN	UMD2
12	WGAC	249A18	chr22:40655172-40778725	no gene	NA	YNN	NYN	YNN	UMD2
13	WGAC	474H10	chr25:40607173-40778447	ACTB	Telomeric	NNY	NNY	YNN	Btau_4.0

*NA: not assigned. We implemented three-letter codes to represent signal patterns detected by FISH or predicted based on Btau_4.0 and UMD2. This is due to the fact that the BAC-FISH method was most reliable to distinguish single signal (the first letter), signals from intrachromosomal (Dup intra, the second letter) or interchromosomal (Dup inter, the third letter) duplications. "Y" means there is while "N" means there is no corresponding signal type. One BAC clone (11L7) had no FISH results.

## Discussion

We present the first detailed genome-wide analysis of recent segmental duplication content of the bovine genome. Global studies of segmental duplication content have become an effective measure to assess one aspect of the quality of whole-genome sequence assemblies [1,51]. Regions of recent segmental duplication remain one of the greatest challenges to finishing a genome assembly. The underlying problem is the same--the correct placement and resolution of large sequence that can be assigned to multiple positions within the genome. An initial assessment of bovine segmental duplication content therefore provides an important level of annotation for the user of genome sequence information in the design and interpretation of future experiments. Moreover, these initial analyses precisely delineate potential regions where whole-genome shotgun or a BAC-enrichment strategy will provide insufficient information for biologists. These regions include gene families important in immunity, digestion, lactation and reproduction traits. The content and structure of these regions will be pivotal to animal evaluation and selection. We therefore propose that such highly duplicated regions be uncoupled from WGS sequencing strategies and be targeted for high-quality BAC-based finishing to resolving their true location, organization, and complexity. The results presented here should provide a framework for the prioritization of such regions.

The detection of recent segmental duplications is sensitive to the quality of the underlying sequence assembly. At least four factors directly impact an assessment of the segmental duplication content within any genome assembly: (1) the depth of sequencing (fold coverage), (2) the methodology of assembly, (3) the quality of common repeat annotation, and (4) level of allelic variation. All of these factors must be taken into account during an assessment of recent segmental duplication content. There are some limitations of this analysis that should be noted. Although many of the expected bovine gene duplications and highly homologous gene families (i.e., cytochrome P450 and lysozme genes) were validated during our analysis, not all were detected. It is clear that duplications have been problematic during sequence and assembly. The analysis of the unplaced chromosome sequence provides the best testament to this effect. The "unplaced" chromosome (ChrUnAll) in Btau_4.0 showed a marked enrichment for blocks of segmental duplication, with almost half (45.2/94.4 Mb) of the duplications assigned to this category.

Despite these methodological and assembly limitations, some important trends regarding bovine segmental duplications emerged during our study. Our bovine segmental duplication estimate is consistent with similar observations in rat [4,4] and dog [8] but lower than human, mouse [1,3,7]. While these differences may be biologically, we suspect that differences in the strategy for genome sequencing and assembly are the most likely cause. The human and mouse genome assemblies are in the "finished" phases combining both clone-based and whole-genome shotgun strategies [7,28]. The duplicated regions represented a major focus in finishing these efforts resulting in a general increase in the amount of duplications as seen in Fig. 4, even when more relaxed cutoffs (10 kb vs. 20 kb) were applied to the dog and bovine genomes. This is because that like rat, the bovine genome is in still in draft version assembled using a hybrid strategy, termed "BAC-enrichment." The BAC-enrichment hybrid strategy entailed low-pass sequencing of individual BAC clones, followed by an enrichment phase where individual WGS reads were mapped to specific BAC projects based on sequence overlap [29,52]. This may also help to explain the unusually large number of unsupported (WGAC-only) duplications.

Our combined experimental and computational results demonstrated that cattle, as a representative of ruminants, is the fourth species whose pattern is reminiscent of the duplication pattern of other mammals (including mouse, rat and dog). Along with rodents and carnivores, these results now confidently establish tandem duplications as the most likely mammalian archetypical organization, in contrast to humans and great ape species which show a preponderance of interspersed duplications. Based on the current Btau_4.0 assembly, bovine recent duplications are distributed in a nonuniform fashion across the genome. In addition to chromosomal differences, we identified 21 duplication blocks (Fig. 2) over 300 kb in length. The majority of bovine duplications are organized as clusters of tandem or inverted intrachromosomal duplications. A similar bias toward clustered duplications was observed in the mouse, rat and dog genome assemblies (Fig. 4) [3-5,7,8]. The molecular basis for this difference in hominoid and other genomes is unknown, although the burst of primate *Alu *retroposition activity ~35 million years ago has been suggested to correlate with the expansion and dispersion of human segmental duplications [35]. Our analyses of the bovine genome also clearly shows a pericentromeric and subtelomeric bias for segmental duplications, indicating that these may be general properties of mammalian chromosomal architecture. An analysis of the evolutionary genetic distance of all segmental duplications as a function of the sum of aligned base pairs (43,597 alignments) showed a bipartite distribution, for intrachromosomal and interchromosomal segmental duplications. Two peaks were observed, at 0.015 substitutions per site (intrachromosomal) and 0.080 substitutions per site (interchromosomal). Assuming a neutral sequence divergence range of 1.9-2.0 × 10^-9 ^substitution/site/year [53], this bipartite distribution may correspond to segmental duplication expansions that occurred relatively recently (~8 and 40 million years ago, respectively).

Sequence analysis between sheep and cattle genes indicated that their divergences ranged between 1.4 and 1.7% at non-synonymous sites and between 6.9 and 7.7% at synonymous sites [54]. Our assessment of the underlying genes reinforces the now relatively commonplace enrichment of specific ontological classes but also identifies lineage-specific genes (> 99.0% sequence identity) potentially important for promoting cattle speciation, adaptation and domestication. At the gene level, for those duplicated genes or gene families in these mammals, both mutation (gene duplication, inactivation, deletion and conversion) and selection (positive and neutral) are implied in lineage-specific adaptations of these mammals to a particular environment. Duplication of genes involved immunity may be particularly important to cattle due to the substantial load of microorganisms present in the rumen of cattle, an increased risk of opportunistic infections at mucosal surfaces and the need for a stronger and more diversified innate immune responses at these locations. For example, *WC1 *genes encode a family of scavenger receptor cysteine-rich (SRCR) proteins found exclusively on γδ T cells in cattle, sheep and swine but not humans or mice [50]. In addition, we found evidence of recent duplication of *ITLN1 *and *SCP2*, which may be involved in iron and lipid transfer in milk. Additional copy of *B2M *in the cattle genome may impact on the abundance of IgG in cow's milk and increase capacity for uptake in the neonatal gut. Previous studies have demonstrated that the lysozyme family has gone through lineage-specific gene amplifications and sequence adaptations to digestion in ruminants including cattle [55-57]. Lysozyme gene duplications were correctly predicted by both *in silico *approaches and independently confirmed by FISH. Although inter- and intrachromosomal FISH signals of 154H9 suggest that that genomic region may be more complex than we currently appreciate, additional sequence analysis and EST expression data provide further support for our observation [29]. This evidence strongly demonstrated that the expansion of the lysozyme gene family is likely essential for both increasing the expression of lysozyme and allowing it to adapt to different functions (immunity vs. digestion) and/or regions (rumen vs. abomasum) of the ruminant digestive system. It is interesting to note that many of the duplicated genes involved in immunity have been adapted to non-immune functions in cattle: e.g. *IFNT*, which is involved in maintaining early pregnancy, and the lysozyme genes, which are involved in digestion [29], agreeing with the "birth-and-death' theory.

Cytogenetics using BAC-FISH can independently test and compare two genome assemblies [58,59]. As our current FISH results were limited and only based on a single Hereford individual, further analysis will be needed to confirm our observations. This could include performing the same FISH experiments in additional unrelated individuals, additional cattle breeds (beef vs. milk) and subspecies (*Bos indicus*), and closely related species like bison, water buffalo and yak. These experiments will help to clarify the effects of inter-individual CNV on our FISH validation. Although copy numbers could not be accurately defined, there were several signs of CNV events in our FISH experiments (such as signal differences between homologous chromosomes for the BAC clones 213C22 and 6B15 at http://bfgl.anri.barc.usda.gov/cattleSD/). It will be also interesting to detect the breed-specific genomic signatures, if any exist, emerged from the intense cattle selection.

Even though our FISH results were not completely definitive, they provided the first preliminary experimental evidence to evaluate the two available bovine genome assemblies, especially in the duplicated regions which are difficult or challenging to assemble. Our results are more consistent with Zimin et al, who reported that significant fewer intrachromosomal duplications (WGAC positive but WSSD negative) were detected in UMD2. However, neither of these two assemblies is perfect in terms of totally agreeing with the FISH results, suggesting a room for further assembly improvement. Another crucial point is that although UMD2 is different from Btau_4.0 and significantly improved in large, high-identity duplicated regions identified only by WGAC, our definition of bovine segmental duplication (union of all WGAC hits with less than 94% sequence identity and WSSD duplication intervals) is essentially assembly independent. This is because our computational approaches (WGAC and WSSD) can effectively detect these local assembly errors and exclude them from subsequent analyses as false positives. In this sense, it is reasonable to believe that if our approaches were applied to UMD2, they would produce a similar estimate of the duplication content. Beyond the 3.1% segmental duplication regions, there are other types of differences between these two assemblies, such as deletions, inversions and translocations. A systematic genome-wide FISH comparison of these two assemblies is beyond the scope of this study but definitely warranted for the future study.

## Additional note

After the completion of this study, a new version of cattle genome assembly UMD3 was made available at ftp://ftp.cbcb.umd.edu/pub/data/Bos_taurus/. Similar *in silico *analyses were also performed on UMD3 for all BACs in Tables 4 and 5, yielding essentially the same results as the analyses reported on UMD2.

## Conclusion

In summary, this study provides insights into the bovine genome evolution and generates a valuable resource for cattle genomics research. We provide a roadmap for improving the quality of specific regions of the cattle genome that will require special care to resolve the copy, content and structure. Duplicated regions will be an important complement to SNP centric genome-wide association studies since SNP discovery and genotyping have been biased against such regions. Characterizing the impact of copy-number and single basepair variation for genes embedded within these regions will be a challenging, next step. Such variation will likely be important in considering the genetic basis of domestication traits and their selection among diverse cattle breeds.

## Methods

### Genome Resources

We downloaded Btau_3.1 and Btau_4.0 genomic sequences from Human genome Sequencing Center at Baylor College of Medicine ftp://ftp.hgsc.bcm.tmc.edu/pub/data/Btaurus/ and whole genome shotgun sequence (WGS) reads from NCBI http://www.ncbi.nlm.nih.gov/. Both cattle genome assemblies were constructed using the BAC-enrichment strategy, which represents a hybrid between whole-genome shotgun sequence and clone-ordered approaches. Btau_4.0 was constructed by adding a small amount of sequence data and sequence scaffolds positioned using the IL-TX radiation hybrid physical map [29]. Genome sequences were derived from the Hereford cow L1 Dominette 01449, (*Bos Taurus*, American Hereford Association registration number 42190680) with an inbreeding coefficient was 31%. The source of the BAC library DNA (CHORI-240) was Hereford bull L1 Domino 99375, registration number 41170496 (Sire of L1 Dominette 01449). UMD2 assembly was downloaded from ftp://ftp.cbcb.umd.edu/pub/data/Bos_taurus/.

### Computational Analysis of Bovine Segmental Duplications

All reported segmental duplication analyses were performed on the Btau_4.0 cattle genome assembly (Oct, 2007). Similar analyses were also performed on an earlier assembly (Btau_3.1). Two different approaches (WGAC and WSSD) were performed as previously described ([1,34]. Whole genome assembly comparison (WGAC) identifies paralogous stretches of sequence through a BLAST-based strategy which depends on the genome assemblies. Using the WGAC approach, we totally identified a total of 129,555 pairwise alignments (≥ 1 kb and ≥ 90% sequence identity) representing putative duplications. High-copy repeat sequences were initially removed using RepeatMasker and a newly constructed cow library of common repeats [29]. Initial seed alignments were ≥ 250 bp and ≥ 88% with repeats subsequently reintroduced to create local alignments. These alignments were then trimmed to better define their end points, and optimal global alignments were performed to generate accurate alignment statistics. As larger, high-identity duplications (≥ 94%) are frequently collapsed within working draft sequence assemblies [28] or may represent artificial duplications within an assembly [34], we compared these WGAC results to whole genome shotgun sequence detection (WSSD) results. WSSD identifies regions (≥ 10 kb in length, ≥ 94% sequence identity) with a significant excess of high-quality WGS reads [1] within overlapping 5 kb windows. We established thresholds based on the alignment of WGS reads against 96 unique cow BACs [53]. BACs were masked for repeats and MegaBLAST alignments of these BACs were performed against a database of WGS reads. We calculated duplication depth by counting the number of WGS reads aligning to 5 kb sliding windows. In addition, we calculated nucleotide divergence between the WGS reads and the BAC sequences for each 5 kb window. The distribution of alignment depth and divergence in this training set allows empirical thresholds to be determined. Consistent with previous studies [1-4,7,8], we define significant alignment depth and divergence scores as those that are greater than 3 standard deviations from the mean. After training, we masked the entire bovine reference genome for repeats with < 10% divergence and all bovine-specific repeat sequences. We then performed MegaBLAST alignments of the WGS reads to the reference genome. Our analysis was based on a comparison of 23,971,214 *Bos taurus *WGS reads against 400 kb segments of the Btau_4.0 assembly. 13,523,039 reads were remapped to the assembly based on the following criteria: ≥ 94% sequence identity; ≥ 200 bp non-repeat-masked bp and at least 200 bp of PhredQ ≥ 30 bp.

Following previous studies [7], we defined segmental duplications based on the union of significant WGAC hits with less than 94% sequence identity and WSSD results (Fig. 1): i.e. WGAC duplication intervals that were greater than ≥ 94% sequence identity and ≥ 10 kb in size but not supported by WSSD, were excluded from the genome-wide calculation of segmental duplications. Paralogous sequence relationships (Fig. 2 and Additional File 1: Fig. S1) were generated using Parasight graphical visualization software [60].The results of Btau_4.0 analyses including pairwise sequence alignment locations, statistics, and gene content are available at http://bfgl.anri.barc.usda.gov/cattleSD/.

### Bioinformatics Analysis of Organization and Gene Contents in Segmental Duplications

Gene content of cattle segmental duplications was assessed using the Glean consensus gene set [29]. Intersections between segmental duplication coordinates and exon positions were compared using mySQL queries. During our analysis, a total of 9,192 Glean genes (from a genome total of 26,700) were identified that had been assigned to duplicated regions. When excluding ChrUnAll, a total of 7,156 Glean genes were identified.

We investigated the genomic distribution of segmental duplications by testing the hypothesis that pericentromeric and subtelomeric regions were enriched for duplications [34]. Since the pericentromeric and subtelomeric regions are not well annotated we defined pericentromeric and subtelomeric regions as 3 Mb from the most centromeric base and 3 Mb from the end(s) of chromosomes, respectively. Since all cattle chromosomes are acrocentric, with the exception of the X chromosome, this results in a 3 Mb pericentromeric region at one end of the chromosome and a 3 Mb subtelomeric region at the other end of the chromosome. In the case of the X chromosome, the pericentromeric region was defined as two 1.5 Mb regions that flank the centromeric region [61] and two 1.5 Mb subtelomeric ends on both ends of the chromosome. No sequence from ChrUnAll was included. All predicted duplicated bases that overlap these regions were totaled and chi-square tests were used to test the null hypothesis of no enrichment as previously described [34]. Repeat analysis and simulation were performed as previously described [4,7].

We obtained a catalog of all bovine peptides from Ensembl ftp://ftp.ensembl.org/pub/current_fasta/bos_taurus/pep/. This yielded 26,271 peptides, 1,160 of which overlap with predicted segmental duplications, and correspond to 826 unique Ensembl genes. PANTHER accessions were assigned to all peptides using the PANTHER Hidden Markov Model scoring tools http://www.pantherdb.org/downloads/. PANTHER accessions with less than five observations among the duplicated genes were not analyzed further. We tested the hypothesis that the remaining PANTHER molecular function, biological process and pathway terms were under- or overrepresented in segmental duplications with the binomial distribution. Bonferroni corrections were used to correct p-values for multiple hypothesis testing. It is worth noting that a portion of the genes in bovine duplication regions may have been annotated with unknown function or have not been annotated, which may influence the outcome of this analysis.

We retrieved the 59 BAC clone sequences based their coordinates on Btau_4.0. We used MegaBLAST to perform sequence similarity search within Btau_4.0 and UMD2, respectively. The blast outputs were manually visualized and compared in parasight [60]. The pattern was roughly assigned as single vs. duplicated or interchromosomal duplications (Dup inter) vs. intrachromosomal duplications (Dup intra).

### FISH and Image Analysis

Forty-six cattle BAC clones from CHORI-240 were selected to validate the predictions of bovine segmental duplications. Additional 13 BAC clones from the same library were used to compare Btau_4.0 and UMD2. Both interphase and metaphase nuclei were prepared using a Hereford smooth muscle cell line isolated from 1 year old male thoracic aorta (AG08501, Coriell Cell Repositories). Metaphase nuclei were examined to identify Dup inter or Dup intra. More intense FISH signals, which localized to a single site, were subsequently examined by interphase nuclei. Interphase analyses were performed in replicates by comparing cells at both G_1 _and G_2 _stages of arrest.

FISH hybridizations were performed as previously described [62]. Briefly, DNA probes were directly labeled with Cy3-dUTP (Perkin-Elmer) by nick-translation. Two hundred nanograms of labeled probe were used for each FISH experiment. Hybridization was performed at 37°C in 2 × SSC, 50% (v/v) formamide, 10% (w/v) dextran sulfate and 3 mg of sonicated salmon sperm DNA, in a volume of 10 μL. Post-hybridization washing was at 60°C in 0.1×SSC (three times, high stringency). Digital images were obtained using a Leica DMRXA epifluorescence microscope equipped with a cooled CCD camera (Princeton Instruments). Cy3 (red) and DAPI (blue) fluorescence signals, detected with specific filters, were recorded separately as grayscale images. Pseudocoloring and merging of images were performed using Adobe Photoshop software.

## Authors' contributions

GEL and EEE conceived and designed the experiments. AC, MV and CL performed FISH analyses. LC, ZC, BZ LKM and JS performed *in silico *prediction and computational analyses. GEL and EEE wrote the paper. All authors read and approved the final manuscript.

## Supplementary Material

Additional file 1 **Supplemental Material file**. Table S1. Ninety-six unique BACs for WSSD threshold calibration. Table S2. Btau_4.0 nonredundant measure of duplicated sequence by chromosome. Table S3. Duplicated gene table. Table S4. Enrichment of molecular function, biological process and pathway terms. Figure S1. Patterns of intrachromosomal and interchromosomal duplication (≥ 5 kb, ≥ 90% sequence identity). Figure S2. Bovine segmental duplications (47%, 45.2/94.4 Mbp) are enriched in the unassigned genome sequence (ChrUnAll). Figure S3. Predominance of tandem segmental duplications. Click here for file
