# Supplementary material for: Analysis of recent segmental duplications in the bovine genome
Source: BMC Genomics. 2009 Dec 1;10:571. doi: 10.1186/1471-2164-10-571 (PMC2796684; doi:10.1186/1471-2164-10-571)

Table S1. Ninety-six unique BACs for WSSD threshold calibration

AC087843.2	AC149763.5	AC150651.3
AC087860.2	AC149769.2	AC150653.5
AC089991.2	AC149774.2	AC150664.2
AC089992.2	AC149778.2	AC150674.4
AC089993.2	AC149779.3	AC150675.2
AC090031.2	AC149782.2	AC150687.3
AC090961.2	AC149783.2	AC150690.7
AC090976.2	AC150482.2	AC150694.2
AC091252.3	AC150488.2	AC150707.3
AC091660.2	AC150492.2	AC150709.3
AC091728.2	AC150499.3	AC150752.4
AC092193.4	AC150512.3	AC150753.3
AC092496.3	AC150515.3	AC150847.5
AC092727.2	AC150516.2	AC150855.6
AC103584.15	AC150517.3	AC150858.4
AC107065.5	AC150524.3	AC150860.4
AC122112.2	AC150530.4	AC150866.2
AC129959.6	AC150532.2	AC150871.4
AC136966.2	AC150540.2	AC150879.3
AC146966.3	AC150542.2	AC150881.4
AC146967.3	AC150544.3	AC150887.4
AC149665.2	AC150546.2	AC150888.5
AC149669.2	AC150548.2	AC150916.3
AC149676.2	AC150561.4	AC150917.2
AC149679.2	AC150569.2	AC150919.6
AC149683.2	AC150573.4	AC150993.4
AC149694.2	AC150574.2	AC151060.4
AC149716.2	AC150577.4	AC151117.4
AC149717.2	AC150593.2	AC151132.3
AC149720.2	AC150635.4	AC156365.3
AC149756.4	AC150644.4	AC156366.3
AC149762.2	AC150650.5	AC156367.3

Table S2. Btau_4.0 nonredundant measure of duplicated sequence by chromosome.

Chromosome		Duplicated Sequence	
name	size	fraction	bases
1	161,106,243	0.55%	890,015
2	140,800,416	0.36%	505,522
3	127,923,604	2.73%	3,496,320
4	124,454,208	1.51%	1,885,311
5	125,847,759	3.39%	4,261,448
6	122,561,022	1.11%	1,357,434
7	112,078,216	1.24%	1,395,058
8	116,942,821	2.08%	2,437,740
9	108,145,351	0.95%	1,030,154
10	106,383,598	2.35%	2,499,539
11	110,171,769	0.87%	957,384
12	85,358,539	1.11%	951,597
13	84,419,198	2.17%	1,832,699
14	81,345,643	1.19%	965,056
15	84,633,453	3.01%	2,548,893
16	77,906,053	1.63%	1,269,471
17	76,506,943	0.90%	686,245
18	66,141,439	4.75%	3,140,374
19	65,312,493	1.82%	1,186,273
20	75,796,353	0.47%	356,560
21	69,173,390	1.96%	1,355,393
22	61,848,140	0.43%	268,420
23	53,376,148	3.01%	1,607,978
24	65,020,233	0.88%	571,147
25	44,060,403	1.71%	752,857
26	51,750,746	1.59%	820,343
27	48,749,334	3.86%	1,880,223
28	46,084,206	0.91%	421,128
29	51,998,940	3.36%	1,748,874
X	88,516,663	6.88%	6,092,914
UnAll	402,224,868	11.25%	45,235,134
TOTAL	3,036,638,192	3.11%	94,407,504
PLACED	2,634,413,324	1.87%	49,172,370

Placed excludes pairwise alignments within chrUnAll.

Table S3. Duplicated gene table

TableS3.xls is available as Additional file 2 or at http://bfgl.anri.barc.usda.gov/cattleSD/.

Table S4. Enrichment of molecular function, biological process and pathway terms

	Genome (23415)	SD (1074)	Expected	Over/Under representation	P-value
**Molecular Function**					
Defense/immunity protein	706	177	32.38	+	5.20E-73
Receptor	2862	277	131.27	+	2.62E-32
Immunoglobulin receptor family member	271	74	12.43	+	3.79E-31
Interferon	41	36	1.88	+	3.80E-31
Other receptor	366	68	16.79	+	2.52E-19
Cytokine	129	37	5.92	+	8.83E-16
Antibacterial response protein	71	28	3.26	+	3.95E-15
Other defense and immunity protein	137	36	6.28	+	3.53E-14
G-protein coupled receptor	1538	128	70.55	+	1.57E-08
Serine protease inhibitor	152	27	6.97	+	1.12E-06
Protease inhibitor	215	32	9.86	+	2.31E-06
Major histocompatibility complex antigen	61	14	2.8	+	2.35E-04
ATP-binding cassette (ABC) transporter	104	17	4.77	+	1.67E-03
Large G-protein	74	13	3.39	+	1.08E-02
Intermediate filament	123	17	5.64	+	1.30E-02
Aspartic protease	143	18	6.56	+	2.56E-02
Nucleic acid binding	2942	51	134.94	-	8.21E-17
Kinase	805	8	36.92	-	1.94E-07
Transcription factor	2096	58	96.14	-	2.53E-04
Protein kinase	614	7	28.16	-	2.79E-04
Extracellular matrix	454	7	20.82	-	1.18E-02
Membrane traffic protein	413	6	18.94	-	1.45E-02
Actin binding cytoskeletal protein	420	5	19.26	-	1.84E-02
Other miscellaneous function protein	479	7	21.97	-	2.91E-02
**Biological Process**					
Immunity and defense	1912	280	87.7	+	4.79E-67
T-cell mediated immunity	349	86	16.01	+	2.95E-33
Macrophage-mediated immunity	207	65	9.49	+	1.24E-30
Interferon-mediated immunity	80	25	3.67	+	2.94E-11
Sensory perception	1457	129	66.83	+	4.48E-11
Olfaction	1063	105	48.76	+	8.26E-11
Chemosensory perception	1073	105	49.22	+	1.06E-10
Other oncogenesis	103	24	4.72	+	3.30E-08
MHCI-mediated immunity	36	14	1.65	+	5.13E-07
Extracellular transport and import	159	28	7.29	+	5.14E-07
Natural killer cell mediated immunity	120	24	5.5	+	6.23E-07
G-protein mediated signaling	1891	143	86.74	+	7.66E-07
Cytokine/chemokine mediated immunity	146	26	6.7	+	1.47E-06
Ligand-mediated signaling	478	53	21.92	+	1.73E-06

Cell surface receptor mediated signal transduction	2797	191	128.29	+	2.40E-06
Cytokine and chemokine mediated signaling pathway	277	36	12.71	+	1.05E-05
Steroid hormone metabolism	58	14	2.66	+	1.62E-04
Protein ADP-ribosylation	19	7	0.87	+	6.93E-03
Proteolysis	1256	87	57.61	+	1.73E-02
Stress response	284	28	13.03	+	2.76E-02
Nucleoside, nucleotide and nucleic acid metabolism	3590	84	164.67	-	2.69E-12
Developmental processes	2390	49	109.62	-	3.05E-10
Protein modification	1318	16	60.45	-	6.29E-10
Cell cycle	1078	15	49.45	-	1.63E-07
Protein phosphorylation	757	7	34.72	-	1.71E-06
Intracellular protein traffic	1138	19	52.2	-	2.12E-06
Ectoderm development	769	12	35.27	-	6.15E-04
Intracellular signaling cascade	1012	20	46.42	-	1.08E-03
mRNA transcription	1972	55	90.45	-	3.05E-03
Cell adhesion	665	14	30.5	-	1.88E-02
Cell structure and motility	1253	35	57.47	-	2.34E-02
**Pathway**					
T cell activation	244	51	11.19	+	2.79E-16

Figure Legends

Figure S1. Patterns of intrachromosomal and interchromosomal duplication (≥ 5kb, ≥ 90% sequence identity). The graphic shows a genome-wide view of interchromosomal (red, with connecting lines) and intrachromosomal (blue bars, with connecting lines) segmental duplications. White bars represent gaps in the genome assembly. For those interchromosomal (red) segmental duplications without connecting lines, their paralogous sequences are localized on ChrUnAll (only shown in Fig. S2). A total of 21 large regions (each ≥ 300 kb in length, total ~12.6 Mb of sequence) are shown as gold bars. For more detail, including sequence identity and pairwise relationships of all duplications and alignments, see http://bfgl.anri.barc.usda.gov/cattleSD/.

Figure S2. Bovine segmental duplications (47%, 45.2/94.4 Mbp) are enriched in the unassigned genome sequence (ChrUnAll). Intrachromosomal (blue) and interchromosomal (red) segmental duplications were displayed as WGAC ≥ 40 kb, ≥ 95% (A) and WGAC ≥ 20 kb, ≥ 95% (B) for simplicity.

Figure S3. Predominance of tandem segmental duplications. Three examples of intrachromosomal tandem duplications (chr15, 18 and X) are shown. Intrachromosomal (blue) and interchromosomal (red) segmental duplications were displayed.

Figure S1

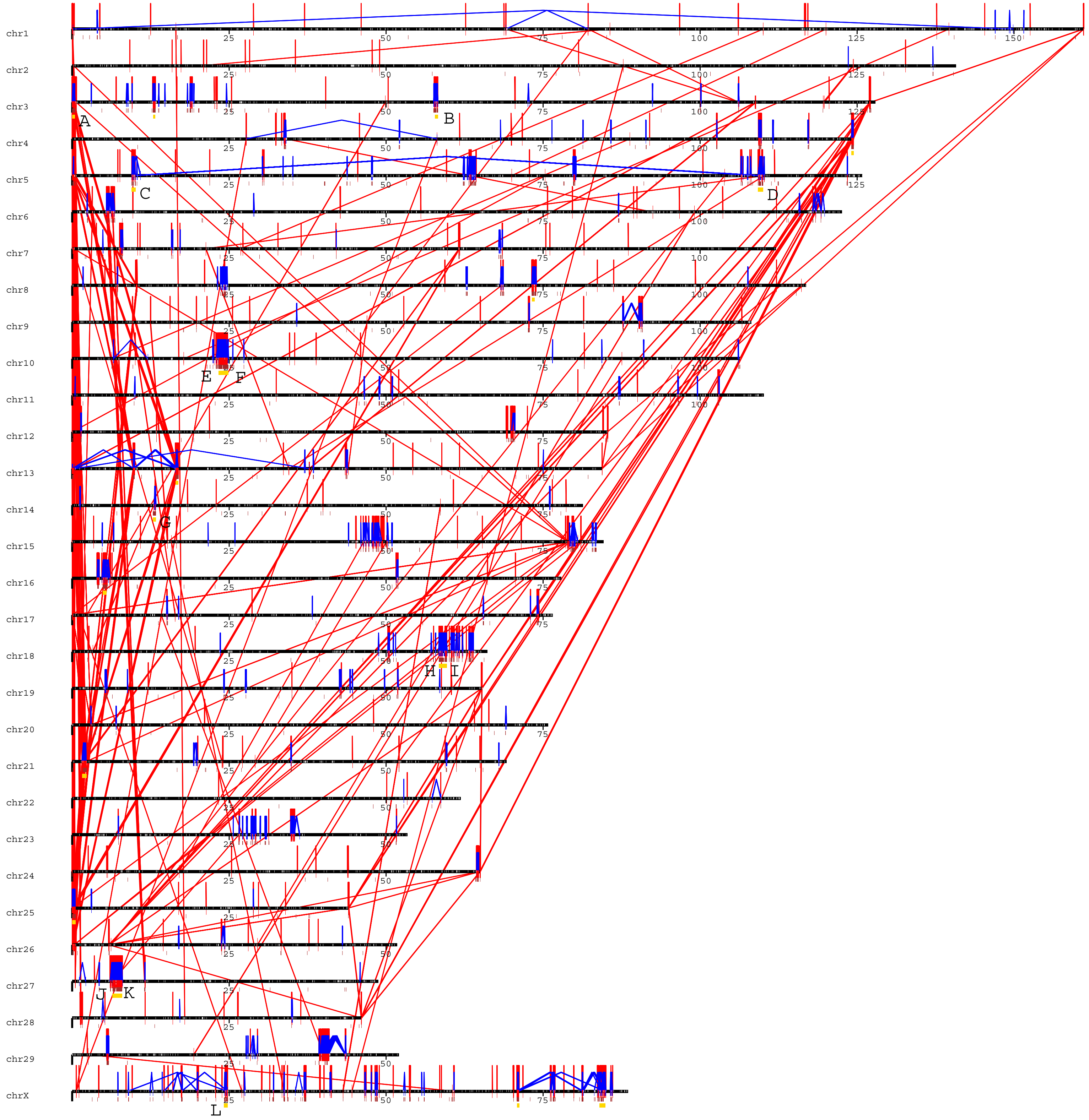

chr1
chr2
chr3
chr4
chr5
chr6
chr7
chr8
chr9
chr10
chr11
chr12
chr13
chr14
chr15
chr16
chr17
chr18
chr19
chr20
chr21
chr22
chr23
chr24
chr25
chr26
chr27
chr28
chr29
chrX
25
50
75
100
125
150
A
B
C
D
E
F
G
H
I
J
K
L

Figure S2

A

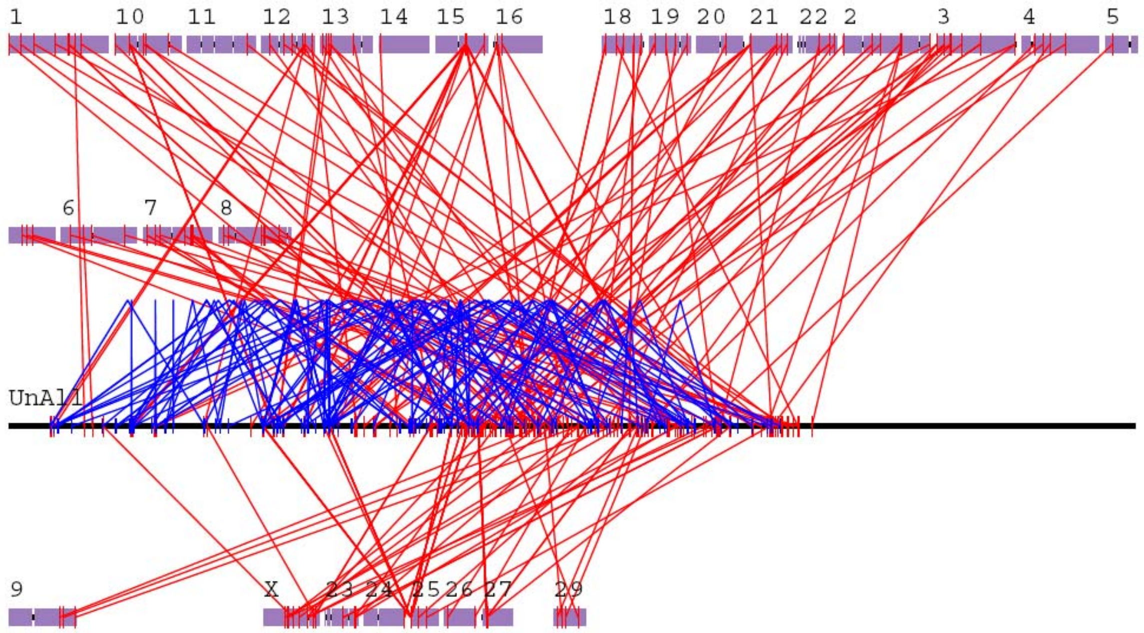
1
10
11
12
13
14
15
16
18
19
20
21
22
2
3
4
5
6
7
8
UnAll
9
X
23
24
25
26
27
29

B

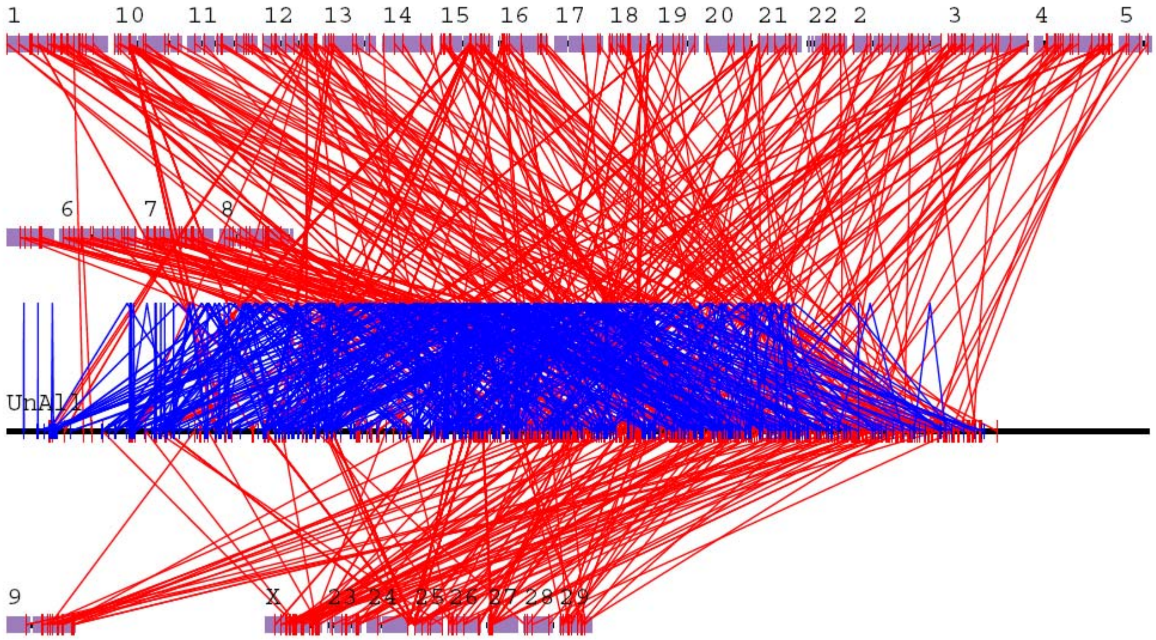
1
10
11
12
13
14
15
16
17
18
19
20
21
22
2
3
4
5
6
7
8
UnAll
9
X
23
24
25
26
27
28
29

Figure S3

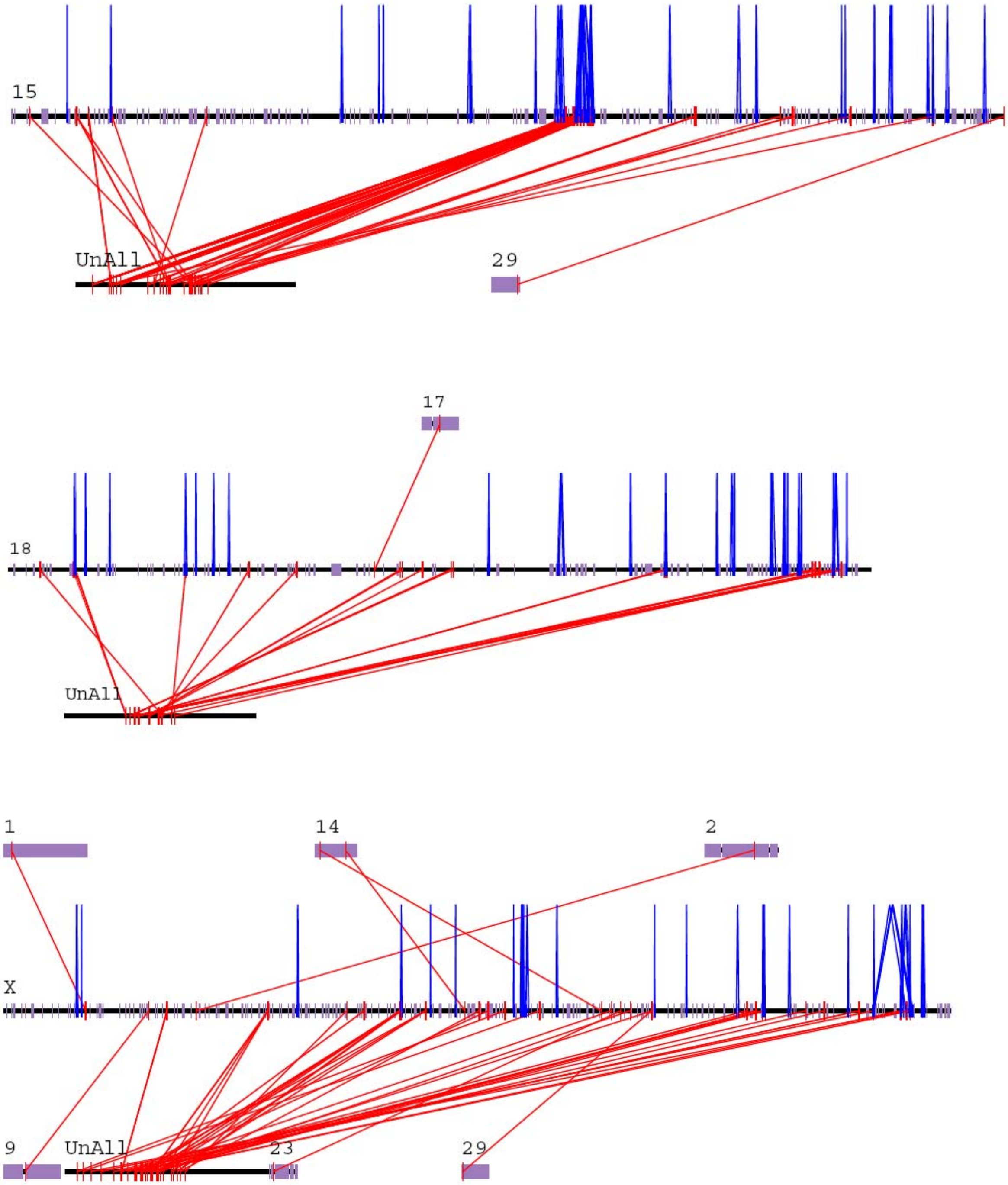

Supplement: Additional file 1 — Supplemental Material file. Table S1. Ninety-six unique BACs for WSSD threshold calibration. Table S2. Btau_4.0 nonredundant measure of duplicated sequence by chromosome. Table S3. Duplicated gene table. Table S4. Enrichment of molecular function, biological process and pathway terms. Figure S1. Patterns of intrachromosomal and interchromosomal duplication (≥ 5 kb, ≥ 90% sequence identity). Figure S2. Bovine segmental duplications (47%, 45.2/94.4 Mbp) are enriched in the unassigned genome sequence (ChrUnAll). Figure S3. Predominance of tandem segmental duplications. [file 1471-2164-10-571-S1.PDF]
